# Metastatic Squamous Cell Carcinoma: A Cautionary Tale

**DOI:** 10.7759/cureus.10879

**Published:** 2020-10-10

**Authors:** Colby Shreve, Chase Shropshire, David G Cotter

**Affiliations:** 1 Dermatology, University of Nevada Las Vegas School of Medicine, Las Vegas, USA

**Keywords:** squamous cell carcinoma (scc), solitary metastasis, cutaneous malignancy, mohs surgery, cutaneous metastasis, metastatic skin cancer, high risk scc

## Abstract

Cutaneous squamous cell carcinoma (cSCC) typically arises from a malignant proliferation of keratinocytes. It is the second most common cancer in the United States and typically affects older white men. Risk factors for cSCC include ultraviolet radiation exposure, light skin tone, and immunosuppression. Although metastasis in cSCC is rare, primary tumor characteristics such as location, size, and depth of invasion, among others, can help risk-stratify lesions for local recurrence, metastatic events, and death. We present a case of primary cutaneous metastatic squamous cell carcinoma masquerading as a cyst on the left temple of a 73-year-old Caucasian man following numerous treatments of cryotherapy to an ipsilateral helical lesion.

## Introduction

Squamous cell carcinoma (SCC) is the second most common type of nonmelanoma skin cancer (NMSC) in the United States, with an estimated annual incidence of over one million and accounting for about 20% of all NMSCs [1,2]. In the United States, the lifetime incidence is estimated to be 7-11%, and the average age of onset is in the sixth decade of life [3,4]. The prognosis for cutaneous SSC after definitive treatment is generally good, with three-year disease-specific survival of around 85% [5].

Metastasis of cutaneous squamous cell carcinoma (cSCC) is rare. However, certain tumor and patient characteristics increase the risk of metastasis. Prior studies have demonstrated metastasis rates of 3-9%, occurring, on average, one to two years after initial diagnosis [6]. We report an insidious presentation of metastatic primary cSCC appearing as a subcutaneous temporal nodule in a 73-year-old Caucasian man.

## Case presentation

**Figure 1:**
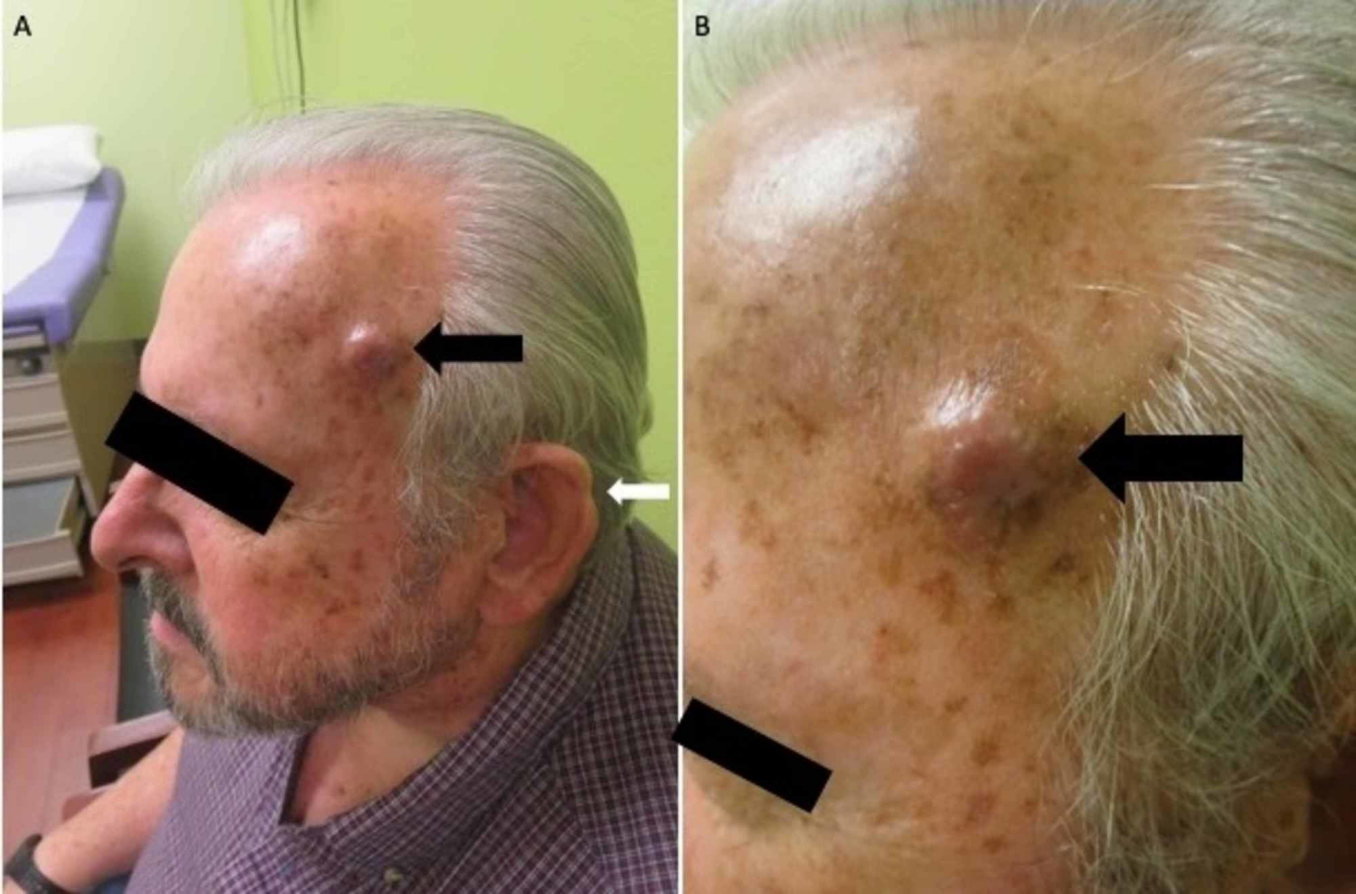
Clinical photographs at initial presentation Panel A shows the left-sided temporal nodule (black arrow) and a cookie-bite type deformity of the left ear (white arrow). Panel B shows a close-up photograph of the left temporal nodule (black arrow).

A 73-year-old man presented to our clinic for a second opinion of an “unsightly cyst” on his left temporal skin (Figures 1A, 1B, black arrow). The lesion had been present for one year and was skin-colored and firm. There were no associated symptoms. He requested removal of this lesion due to its appearance, and he had been turned away by others in the past due to the perception that such a procedure would be “cosmetic” in nature. His medical history was negative for skin cancer. However, review of records from the prior clinician revealed that a clinically diagnosed “pre-cancer” of the ipsilateral helical rim had been repeatedly treated with cryotherapy using liquid nitrogen, finally resulting in keloid formation. Ultimately, his left helical rim keloid was removed by shave technique, which resulted in a cookie-bite type deformity of his left ear (Figure 1A, white arrow).

In our clinic, he underwent a diagnostic excision of the left temporal lesion. The excision failed to reveal a circumscribed cyst. Rather, an ill-defined and friable subcutaneous mass had been excised.

**Figure 2:**
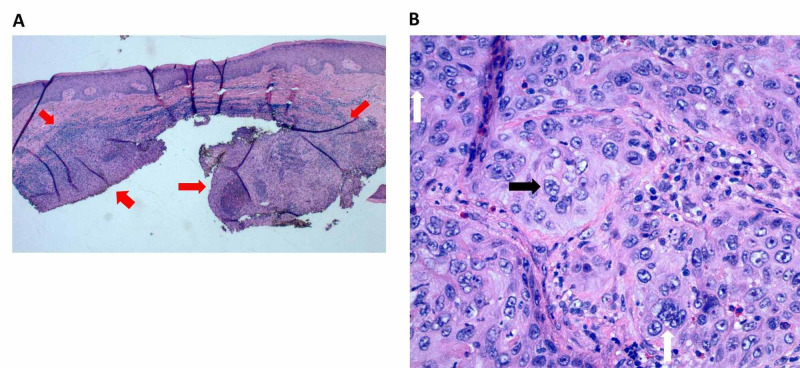
Histopathology slides of the biopsy site (hematoxylin and eosin stain) Panel A shows a dense collection of keratinocytes with host-mediated inflammation (red arrows) that notably spare the epidermis. Higher magnification shown in panel B reveals cells with nuclear atypia (white arrows) and mitoses (black arrow).

**Figure 3:**
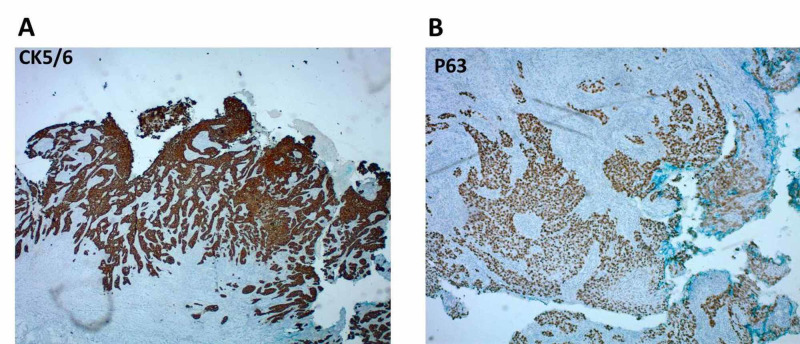
Immunohistochemical staining confirm primary cutaneous metastatic squamous cell carcinoma Immunohistochemical staining with cytokeratin 5/6 (CK5/6; panel A) confirmed keratinocyte lineage. Further immunostaining with p63 confirmed primary cutaneous origin of these malignant keratinocytes (panel B).

Microscopic examination of the tissue revealed a poorly circumscribed collection of highly pleomorphic and mitotically active cells within the dermis that notably lacked any epidermal connection (Figures 2A, 2B). Immunohistochemical staining with cytokeratin 5/6 confirmed keratinocytic lineage, and p63 staining confirmed a primary cutaneous origin of the malignant cells (Figures 3A, 3B).

**Figure 4:**
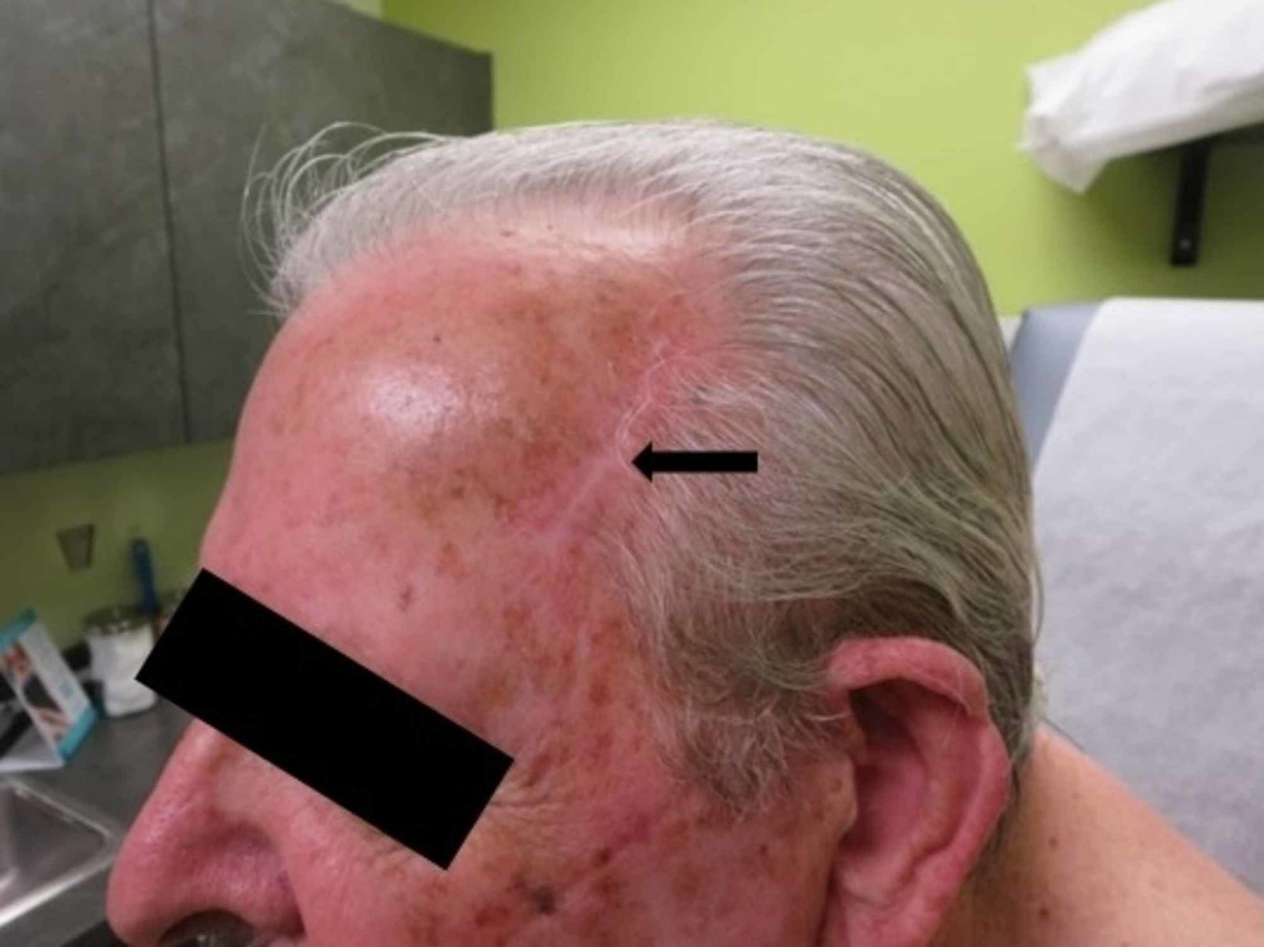
Clinical photograph at 11 months follow-up Follow up examination at 11 months post-Mohs surgery shows a well-healed scar (black arrow) and no signs of recurrence

These findings confirmed a diagnosis of primary cutaneous metastatic SCC. Following diagnosis, he underwent full-body skin examination, lymph node examination, and positron emission tomography and computed tomography (PET-CT) of the chest, abdomen, and pelvis, in search of a primary lesion and/or other metastatic disease. No primary SCC was identified. Also, additional metastatic SCC was not observed. His left temple metastasis was fully extirpated through Mohs micrographic surgery (MMS). At 11 months follow-up, he remains cancer-free and shows no signs of recurrence (Figure 4).

Since our patient did not have a prior history of skin cancer and since no other cutaneous or systemic SCC was discovered, we speculate that his left helical rim "pre-cancer" may have indeed been an SCC that subsequently metastasized to his temple.

## Discussion

The risk of cSCC metastasis is low and has been cited to range between 35 and 9% [6]. When metastasis does occur, the vast majority of metastases are found in the parotid or cervical lymph nodes. In a review of metastatic cSCC in England from 2013 to 2015 by Venables et al., the most common site of metastasis was the parotid or cervical lymph nodes (73.6%) followed by axillary nodes, groin nodes, and distant metastasis [7]. The primary site with the highest risk of metastasis is the ear [7,8].

Although metastasis in cSCC is rare, certain features can estimate the likelihood of metastasis. High-risk features include diameter greater than 2 cm, depth of invasion greater than 4 mm or through subcutaneous fat, perineural involvement, poor differentiation on histology, location on high-risk areas such as the lip or ear, tumor recurrence, and an immunocompromised state [9-11]. These features have been established by the American Joint Committee on Cancer, National Comprehensive Cancer Network, and Brigham and Women’s Hospital, and are summarized in Table 1.

**Table 1:** High-risk tumor and patient characteristics AJCC, American Joint Committee on Cancer; NCCN, National Comprehensive Cancer Network; BWH, Brigham and Women’s Hospital Breslow depth is the actual measurement of tumor invasion in millimeters. Clark's level describes the level of anatomical invasion of cancer in the skin. Level I is confined to the epidermis. Level II indicates invasion into the papillary dermis. Level III indicates invasion to the junction of the papillary and reticular dermis. Level IV indicates invasion into the reticular dermis. Level V indicates invasion into the subcutaneous tissue. "Mask region" of the face: central face, chin, ear, eyebrow, eyelid, lip, mandible, nose, periorbital, preauricular, postauricular, temple.

AJCC	NCCN	BWH
>2-mm Breslow depth	Location on “mask region” of face	Tumor diameter > 2 cm
Clark’s level > IV	Poorly defined borders	Poorly differentiated
Location on hair-bearing lip and ear	Site of prior radiotherapy or chronic inflammatory process	Perineural invasion > 0.1 mm
Perineural invasion	Immunosuppression	Tumor invasion beyond fat
Poorly differentiated	Recurrent tumor	
	Rapidly growing	
	Neurologic symptoms	
	Poorly differentiated	
	Adenoid, adenosquamous, desmoplastic, or metastatic subtypes	
	Depth > 2 mm or Clark’s level IV or V	
	Perineural, lymphatic, or vascular invasion	

Management of cSCC depends on the presence of high-risk features. In low-risk cSCC, standard surgical excision is generally acceptable. In tumors < 2 cm, surgical excision with 4-mm margins provides clear margins in 95% of cases [9,10]. In high-risk cSCC, MMS is the treatment of choice. MMS allows for visualization of all margins of the specimen in real time, thus ensuring that the tumor is clear prior to closure. This provides a strong therapeutic advantage in high-risk cSCC, as these tumors may be deeper or more infiltrative. Studies have demonstrated that MMS is highly effective in treating both primary and recurrent high-risk cSCC, with cure rates of 97% and 94%, respectively [9,10].

All patients with suspected or diagnosed cSCC should undergo regional lymph node examination and those diagnosed with high-risk cSCC should undergo CT or PET scan to assess for metastasis [9]. Treatment for metastatic cSCC is similar to primary lesions and includes surgery, radiotherapy, and chemotherapy. The presence of distant metastases is an indication for chemotherapy, whereas regional metastases are treated with surgery or radiation or a combination of the two. Veness et al. demonstrated that patients undergoing combined therapy had lower rates of recurrence (20% vs. 43%) and a higher five-year disease-free survival rate (73% vs. 54%) than surgery alone [11]. Furthermore, targeted therapies, including EGFR inhibitors, PD-1 inhibitors (cemiplimab), and p53 inhibitors, are and continue to emerge as novel and effective therapies for cSCC [9,12].

## Conclusions

Although relatively rare, metastatic cSCC is potentially deadly. Although no primary lesion was ever identified in our patient, it is possible that the ipsilateral left helical lesion that had been treated repeatedly with cryotherapy was the source of his metastatic cSCC. It is also possible that the SCC arose from the cyst itself, as they can also express p63. These cases are rare but possible in the setting of irritation and inflammation. This case demonstrates the danger of using multiple rounds of cryotherapy on a recurrent helical lesion and emphasizes the importance of biopsy of suspicious and recalcitrant lesions. Furthermore, had the patient been reassured that his temporal lesion was simply a cyst, it would have grown and likely metastasized to other parts of the body, resulting in additional treatment and a higher chance of mortality.
